# Photochemical Copper Coating on 3D Printed Thermoplastics

**DOI:** 10.1038/srep31188

**Published:** 2016-08-09

**Authors:** Winco K. C. Yung, Bo Sun, Junfeng Huang, Yingdi Jin, Zhengong Meng, Hang Shan Choy, Zhixiang Cai, Guijun Li, Cheuk Lam Ho, Jinlong Yang, Wai Yeung Wong

**Affiliations:** 1 Department of Industrial and Systems Engineering, The Hong Kong Polytechnic University, Hung Hom, Hong Kong, HKSAR; 2 School of Reliability and Systems Engineering, Beihang University, No. 37 Xueyuan RD. Haidian, Beijing 100191, China; 3 Hefei National Laboratory for Physical Sciences at Microscale, University of Science and Technology of China, Hefei, Anhui 230026, China; 4 Institute of Molecular Functional Materials and Department of Chemistry, Hong Kong Baptist University, Waterloo Road, Hong Kong, HKSAR; 5 Department of Applied Biology and Chemical Technology, The Hong Kong Polytechnic University, Hung Hom, Hong Kong, HKSAR

## Abstract

3D printing using thermoplastics has become very popular in recent years, however, it is challenging to provide a metal coating on 3D objects without using specialized and expensive tools. Herein, a novel acrylic paint containing malachite for coating on 3D printed objects is introduced, which can be transformed to copper via one-step laser treatment. The malachite containing pigment can be used as a commercial acrylic paint, which can be brushed onto 3D printed objects. The material properties and photochemical transformation processes have been comprehensively studied. The underlying physics of the photochemical synthesis of copper was characterized using density functional theory calculations. After laser treatment, the surface coating of the 3D printed objects was transformed to copper, which was experimentally characterized by XRD. 3D printed prototypes, including model of the Statue of Liberty covered with a copper surface coating and a robotic hand with copper interconnections, are demonstrated using this painting method. This composite material can provide a novel solution for coating metals on 3D printed objects. The photochemical reduction analysis indicates that the copper rust in malachite form can be remotely and photo-chemically reduced to pure copper with sufficient photon energy.

3D printing of thermoplastics has been explosively growing in recently developed markets for modelling, prototyping and production applications^1^,^2^,^3^. Among all the 244,533 shipments of 3D printers in the year 2015, 97.5% of them belonged to the fused deposition modeling (FDM) type which is based on extrusion of melting thermoplastics layer by layer; and this number was anticipated to rise up to 5.6 million in 2019^4^. Given such a huge number of 3D printer shipments, there will be billions of 3D objects printed in the near future. In addition, the metal coating on the surface of these 3D printed objects is versatile. Aesthetically, metals with surface finishing have a more glamorous luster than that of plastics^5^, and for the wear resistance, the metal surfaces are more durable than plastics^6^. As for heat dissipation, metals possess significantly higher thermal conductivity than plastics^7^. In addition, selectively patterned metals can also be utilized as electronic circuits^8^, as well as antennas^9^ and RFID^10^. For example, copper nanoparticles were successfully patterned into circuits over larger areas using inkjet printing on flat surfaces with a close nozzle-to-substrate distance^11^. However, it is challenging to directly coat the surface of 3D printed thermoplastics with metal. Thermoplastics are naturally insulating, so it is impossible to directly electroplate on their surfaces^12^. Due to their low melting point, it is also impractical to directly cast metals on 3D objects without damaging their original shapes^13^. The laser induced forward transfer is therefore not suitable to be applied in 3D printing for substrates with lower operation temperatures^3^. Thus there is strong demand for a novel method to coat metals on an emerging number of 3D printed thermoplastics without costly and specialized facilities.

For surface coating, painting is one of the most exhaustively used methods to coat functional materials onto base objects such as aesthetic pieces and house furniture^14^. As a historic paint materials, malachite has been used since ancient Egyptians time^15^. This material has also been long established for breaking down into several separate elements, as the first experimental demonstration of the law of constant composition^16^. However, these synthesized copper components are in the form of black copper oxides, rather than metallic copper, making the reaction production less useful than in their metal phases. In addition, the native copper can also be transformed into malachite after a long period of time, and is frequently observed in copper based antiques, such as the Statue of Liberty in New York^17^. Although electrochemical methods can be used to reduce the malachite into its native copper, this contact-mode method would result in contaminants as well as potential damage to valuable antiques. Photochemical reaction is a remote and non-contact method for reducing metals and the UV irradiation was previously investigated for synthesizing copper from Cu_2_(OH_2_)_2_(O_2_C(CH_2_)_4_CH_3_)_4_^18^.

Herein, we have demonstrated that malachite, which is one of the major compositions of the surface composition of the Statue of Liberty in New York City^19^, can be transformed to copper by non-contact laser treatment. Further malachite mixed in acrylic paint can be used for coating on 3D printed objects and later transformed to copper after one-step laser treatment. Acrylic paint has been important in art and house painting since it was invented in 1934, due to its fast-drying and waterproof features^20^. By dissolving acrylic paint in ethyl acetate, malachite can be used in a similar way to commercial acrylic paints. Acrylic paint containing malachite is shown in Fig. 1(a), together with seven commercial acrylic paints in red, orange, yellow, green, dark blue, indigo, and dark violet colors. With an optimized mass ratio of malachite to acrylic at 5:2, the mixture is sticky enough for coating on printed polylactic acid (PLA) surfaces and its coating is thick enough for subsequent laser treatment.

## Results and Discussions

**Figure 1:**
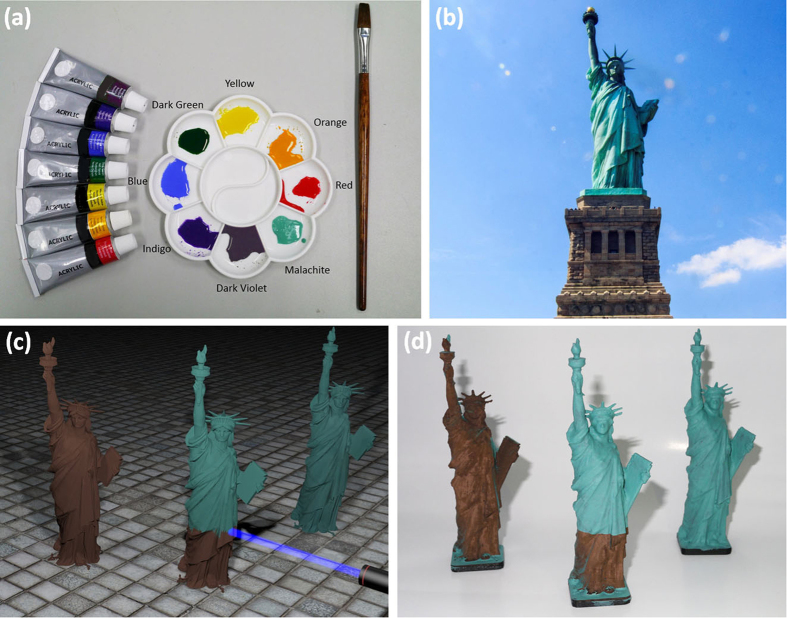
Malachite coating for the Statue of Liberty model. (**a**) The acrylic paint containing malachite together with different commercial acrylic paints. (**b**) The actual Statue of Liberty in New York City (taken by Dr. Youxi Lin from Stony Brook University, New York). (**c**) Whole process in computer simulation. (**d**) The 1:600 models fabricated using the acrylic paint containing malachite and the models after the laser treatment.

As a demonstration, a 1:600 scale model of the Statue of Liberty was designed using Autodesk 3Ds Max software according to the original statue in New York City in Fig. 1(b). After prototyping the 1:600 ratio PLA-based models using a FDM 3D printer, the acrylic paint containing malachite was brush-coated on the surface. After the paint on the surface of the 3D models was dried, a 405 nm laser was scanned on the coating line by line. The coating area on the 3D printed thermoplastic models was photochemically transferred from malachite to copper after laser writing, and the overall workflow is shown in Fig. 1(c). Following these fabrication sequences, the prototypes were built as shown in Fig. 1(d). The coating area without the laser treatment was in malachite green color and the area after the laser treatment was a natural bronze color, similar to the real Statue of Liberty, as shipped to New York City in 1886^21^. From right to left, the models of the Statue of Liberty before, during and after laser treatment are shown respectively. It is proven that copper can be coated on the surfaces of 3D printed thermoplastics using these two-step processes of painting and post laser treatment.

**Figure 2:**
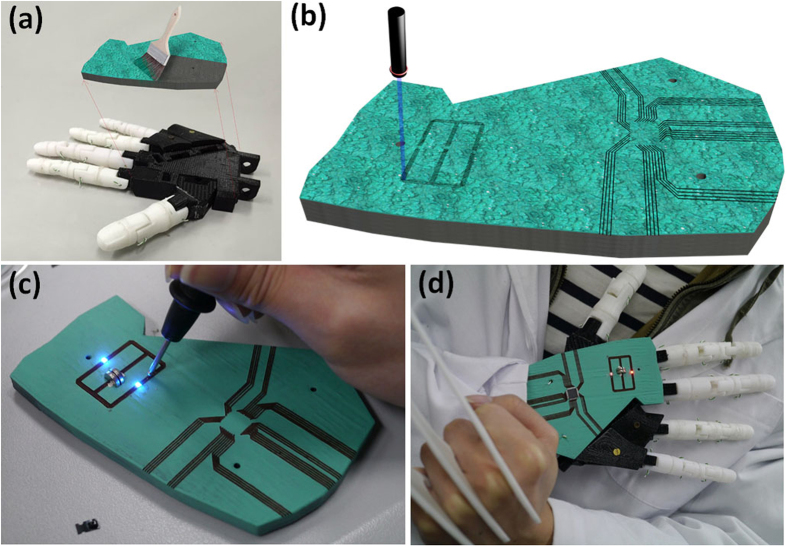
Selective laser patterning on the acrylic paint containing malachite. (**a**) One part of a 3D printed robot hand was brush-coated with the acrylic paint containing malachite. (**b**) Laser writing tracks for patterning the interconnection circuits on the acrylic paint containing malachite. (**c**) Assembly and testing of the synthesized circuits for powering up blue LEDs. (**d**) The robot hand with on-chip batteries, red LEDs and microprocessors using malachite coating and laser patterning.

Apart from the transformation of the whole coating from malachite to copper, the selective laser writing can also pattern conductive copper tracks on the 3D printed thermoplastics coated with malachite. As an illustration, the electronic circuits for powering two LEDs using on-chip batteries and for wiring up microchips were fabricated, as shown in Fig. 2. A robot hand of normal human hand size was printed with PLA using a FDM 3D printer. The acrylic paint containing malachite was also brush-painted on the top surfaces of the robot hand as in Fig. 2(a). Two series of interconnection circuits were then patterned by direct laser writing on the sample surface, as shown in Fig. 2(b). After the laser writing process, the LEDs connected in parallel were mounted on the circuits together with the on-chip batteries. The voltage across the LEDs measured by multimeter was 2.99 V, as in Fig. 2(c), which is close to the original on-chip battery voltage in series, i.e., 3 V. Thus it can be inferred that this fabricated circuit can efficiently power up on-chip LEDs. After assembling these parts, the robot hand was integrated with these LEDs and microchips, as demonstrated in Fig. 2(d). Thus this one-step laser treatment can efficiently pattern copper on both flat and curved surfaces with the acrylic paint containing malachite, which can be seen in the attached movie.

The above results show that acrylic paint containing malachite can be photochemically transformed to conductive copper by laser. However, the physics behind this mechanism has not been studied before, so the corresponding theoretical calculation and experimental studies of the process were undertaken.

**Figure 3:**
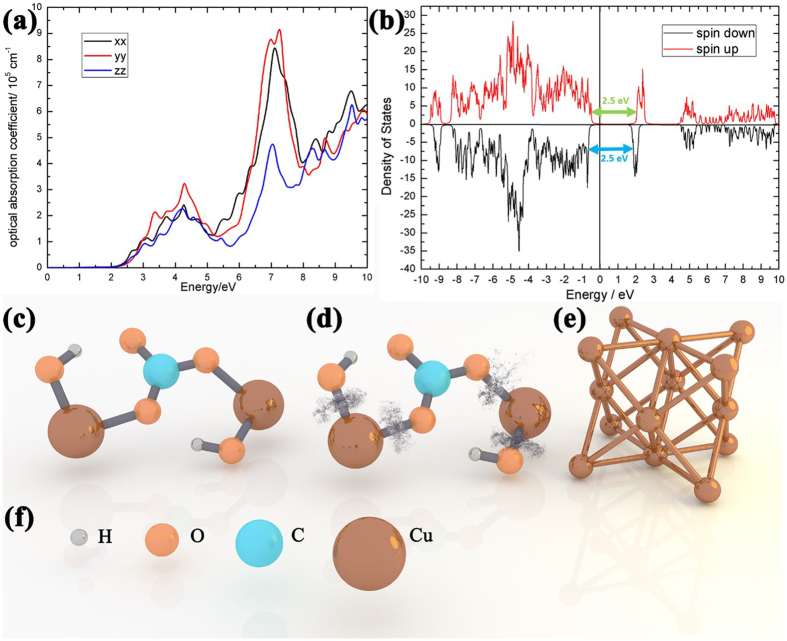
DFT results and molecular structures. (**a**) The calculated optical absorption coefficient according to different incident photon energies. (**b**) Density of states of malachite calculated using spin-dependent density functional theory. (**c**) The pristine malachite molecular structure. (**d**) The bond-broken malachite molecule structure after 405 nm laser treatment. (**e**) The photochemically synthesized copper with an fcc crystalline structure. (**f**) The notion of the individual element for the above illustration figures.

The optimized ionic structure of malachite is depicted in Fig. 3(c). The copper ions (small size, in blue) are only connected with oxygen ions (large size, in red). So if the photon energy is sufficiently high, the copper(II) atoms can be cleaved from the copper-oxygen bonds and form copper(0), as a homolysis process^22^. In order to efficiently trigger this photochemical reaction, two conditions have to be simultaneously satisfied. The first condition is that the system can absorb photons with sufficient efficiency. The optical absorption coefficient is calculated using the density functional theory according to different photon energies, as shown in Fig. 3(a). With a photon energy of 3.06 eV, the absorption coefficients in all directions are up to an amplitude of 10^5 ^cm^−1^. Also, the density of states of the malachite with different spin polarization is calculated with density functional theory (DFT) as shown in Fig. 3(b). There are no states observed in the fermi levels of both the spin up and spin down configurations, indicating the malachite as a semiconductor. The bandgap can be derived as 2.5 eV from the states between the fermi levels. As a result, the photon absorption according to this bandgap should be sufficiently high. Thus the absorption ratio at 405 nm should be large enough for triggering a photochemical reaction. At the same time, the second condition for triggering this photochemical effect is that the photon energy should be greater or equal to the bond dissociation energy of the copper-oxygen bond, because only one photon is responsible for each photochemical reaction, as determined by the Stark Einstein law. The bond dissociated energy is approximately 2.99 eV^23^ while the photon energy of the 405 nm laser is around 3.06 eV. The photons at the 405 nm wavelength can cleave the copper-oxygen bond accordingly. Satisfying both the above two conditions, photons from the 405 nm laser with sufficiently high energy can facilitate breaking of the copper-oxygen bond through homolysis process, as shown in the illustration in Fig. 3(d). Heating can also break these chemical bonds, however, the heated copper at ambient conditions can be easily oxidized, resulting in the formation of copper oxide. If the laser energy is too high, the resultant heat can oxidize the synthesized copper. If the laser energy is too low, the malachite may not be efficiently transferred to copper. This explains why the 405 nm photons in the sunlight spectra did not transfer the Statue of Liberty back to copper. Nevertheless, the composition of the synthesized materials herein remains unknown and is required to be experimentally characterized.

**Figure 4:**
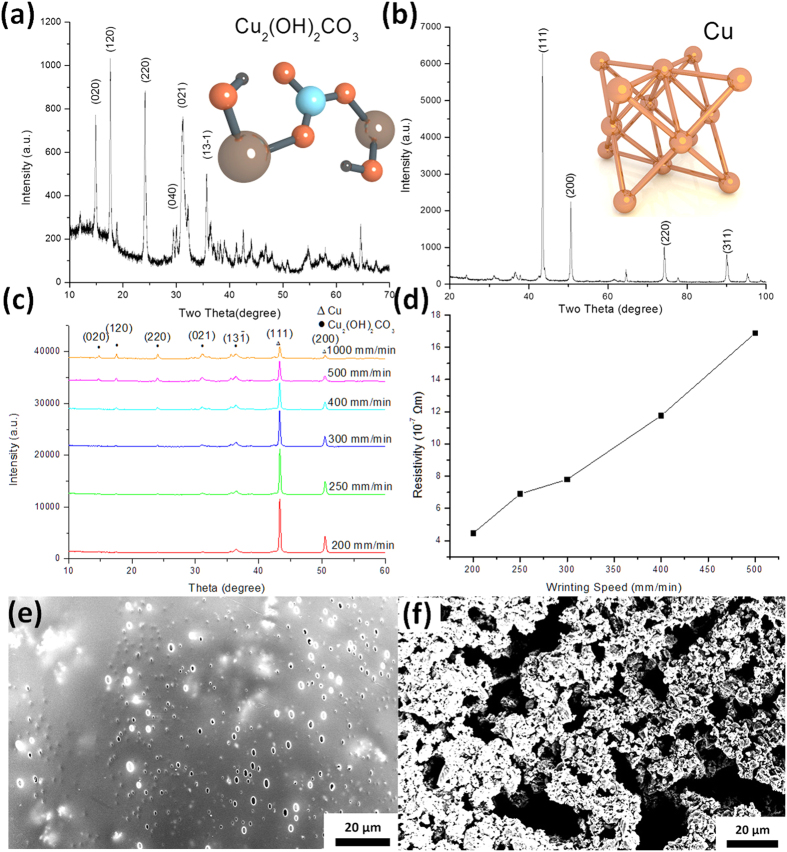
XRD of the acrylic paint containing malachite (**a**) before laser treatment, (**b**) after laser treatment, (**c**) and according to different laser writing speeds. (**d**) Resistivity according to different writing speeds. SEM plan-view images of the surface of acrylic paint containing malachite (**e**) before and (**f**) after laser treatment.

To experimentally verify their compositions under the influence of a laser, XRD analysis was performed on the malachite samples before and after the laser treatment, as shown in Fig. 4. Before the laser treatment, the as-prepared sample behaves like a typical malachite with monoclinic prismatic crystalline structures (JCPDS-ICDD 41–1390), as in Fig. 4(a). After the laser treatment, however, all the peaks characterizing the malachite in the sample disappeared as shown in Fig. 4(b). Instead, the peaks (JCPDS-ICDD 04–0836) characterizing copper with a dominant face centered cubic (fcc) arrangement are observed in the laser treated sample. In this way, it can be inferred that the 405 nm photons transformed the semiconducting malachite back to metallic copper. However, it cannot explain why the 405 nm photons within sunshine spectra did not turn the real Statue of Liberty in New York back to copper. We thus performed a detailed XRD analysis after laser illumination of different writing speeds, as in Fig. 4(c). Given the relative peak intensities of copper and malachite, the average photon energy is inversely proportional to the product yields of copper from malachite. The amount of these two phases can be evaluated using their intensity ratios as a relative order parameters in equation (1)^24^:


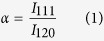


where I_111_ and I_120_ correspond to the copper (111) peak and malachite (120), respectively. As the laser writing speed decreases from 1000 to 200 mm/min, the order parameter increases significantly. So the average intensity illuminated by the laser on the malachite is crucial for the final product composition. For writing speeds lower than 200 mm/min, the synthesized copper ratio is lower, probably due to the heating from the over-absorbed photon energies. For the coating with a 5:2 malachite to acrylic mass ratio, 200 mm/min was found to be the optimized parameter for transforming the composites to copper. The resistances according to different laser writing speeds were characterized using four-probe measurement and the results were plotted in Fig. 4(d). The resistance decreases linearly as the laser writing speed slows down from 500 to 200 mm/min. This linear relation should be ascribed to the copper amount increase with a slower laser writing speed, which is consistent with the XRD results in Fig. 4(c). Meanwhile, it could be noted that the measured resistance was higher than that of the bulk copper (1.68 × 10^−8^ Ωm) even with optimized laser parameters (4.3 × 10^−7^ Ωm). The underlying reason may be due to the porous microstructures, formed by the gas emitting during the laser treatment process as shown in the SEM images of Fig. 4(e,f). Before laser treatment, the surface of acrylic paint containing malachite was smooth as prepared by doctor blade coating. However, the surface became highly porous as shown in Fig. 4(f). The voids formed between the copper clusters may be the reason that the synthesized copper had lower conductivity than that of bulk copper.

Given the ease of applying acrylic paint containing malachite, this coating can apply copper on various 3D printed non-conductive objects without using any expensive facilities. We endeavor this low-cost and easily applied technique will be widely adopted by DIY lovers for broad applications. In addition, with the aid of the photochemical reduction of malachite to copper using the 405 nm laser, it is possible to remove rust from copper antiques. Considering the wide application of drones as unmanned aerial vehicles and robotic vehicles, it is feasible that someday the drones carrying a 405 nm laser can be used to non-contact reduce the real Statue of Liberty in New York, revealing its original color from over 100 years ago.

In comparison with the conventional precursors for coating onto the thermoplastics, applying malachite is better than using copper powders. For the direct application of copper, the separated copper needs to be heated to melting temperatures^3^. However, the absorbed heat can induce huge damage on the underlying thermoplastics shapes. Meanwhile, the malachite precursor can produce copper at much lower temperatures through photochemical rather than thermal effects. So it is more favorable for its deposition on thermoplastics compared to the powders. In addition, the laser diode used here is more affordable than high power lasers, benefiting the users without access to expensive laser systems. For the remaining malachite without laser treatment, it is challenging to remove it with solvents. Nevertheless, the remaining malachite would typically not influence the conducting performance of the circuits since malachite is wide bandgap semiconductor.

Summarizing, a novel acrylic paint containing malachite was introduced for coating on 3D printed objects, and can be transformed to copper via one-step laser treatment. The malachite containing pigment can be used as a commercial acrylic paint, which can be brushed onto 3D printed objects. The material properties and photochemical transformation processes were comprehensively studied. The underlying physics of the photochemical synthesis of copper was characterized using DFT calculations. After the laser treatment, the surface coating of the 3D printed objects was transformed to copper, which was experimentally characterized by XRD. Taking advantage of the computer-aided design, the copper can either be selectively patterned or fully transformed to conductive copper after the laser writing process. 3D printed prototypes, including a Statue of Liberty model covered with a copper surface coating and a robot hand with copper interconnections, are demonstrated using this painting. This composition material can provide a novel solution for coating metals on 3D printed objects. The photochemical reduction analysis indicates that the rust copper in the malachite form can be remotely and photo-chemically reduced to copper with sufficient photon energy.

## Methods

### Computational

Geometry optimization and optic calculations of malachite were performed by using the spin polarized density functional theory (DFT) plus the effective Coulomb interaction (U) (DFT + U) formalism method implemented in the Vienna ab initio Simulation Package (VASP)^25^. The exchange-correlation energy was computed by using the Perdew-Burke-Ernzerhof (PBE) function. The ion-electron interaction was solved with the projector-augment-wave technique^26^,^27^. For geometry optimization, both the lattice constants and atomic positions were relaxed until the forces on the atoms were less than 0.02 eV/Å and the total energy change was less than 1.0 × 10^−5^ eV. A 3 × 3 × 7 Monkhorst-Pack^28^ grid and a kinetic energy cut-off of 500 eV were selected and the U value was 7.12 eV^29^. For static and optical calculations, a finer 12 × 12 × 28 grid was chosen. The wavelength-dependent dielectric function was calculated, and then the absorption coefficient as a function of photon energy was evaluated according to the equation (2):


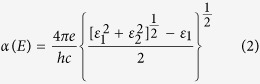


### Materials

The malachite and acrylic powders were used as purchased from Alfa Aesar (CAS: 12069-69-1) and Mitsubishi (model VH001), respectively. 5 g malachite and 2 g acrylic powders were dispersed in 100 mL ethyl acetate, and the mixture was stirred until the acrylic was fully dissolved. Then the acrylic paint containing malachite was brush-painted onto the 3D printed PLA objects.

### Characterization

The crystalline structures of malachite before and after laser treatment were studied with a Bruker D8 Advance X-ray Diffractometer (XRD) system. In order to achieve constant and repeatable measurement for the XRD characterization, the thickness of the malachite in the acrylic paint was fixed at 50 μm, controlled by the doctor-blade. The material used in a FDM 3D printer was commercial PLA of 1.75 mm diameter. The infill rate was set at 10%. Each layer thickness was fixed at 0.27 mm with a feed speed of 41 mm/s.

### Laser writing system

A simple laser writing system was constructed. A 405 nm GaN laser diode was mounted on a tailor-made X-Y moving system driven by 2 stepper motors, with the output power fixed at 0.4 W. The circuit patterns were designed with Autodesk AutoCAD software. Each line represented a width at 50 μm. The CAD files were then transformed to G-code using open-source GRBL firmware and loaded to an open-source Arduino microcontroller. The speed of the laser writing was fixed at 10 mm/min. The laser beam was focused onto the donor layer and the laser beam size was measured to be 200 μm.

## Additional Information

**How to cite this article**: Yung, W. K. C. *et al.* Photochemical Copper Coating on 3D Printed Thermoplastics. *Sci. Rep.* **6**, 31188; doi: 10.1038/srep31188 (2016).

## Supplementary Material

Supplementary Movie S1

Supplementary Information
