# Supplementary material for: Photochemical Copper Coating on 3D Printed Thermoplastics
Source: Sci Rep. 2016 Aug 9;6:31188. doi: 10.1038/srep31188 (PMC4977584; doi:10.1038/srep31188)
Supplement: Supplementary Information [file srep31188-s2.pdf]

Supporting Information for

Photochemical Copper Coating on 3D Printed Thermoplastics

Winco K. C. Yung[1]†, Bo Sun[1,4]†, Junfeng Huang[1]†, Yingdi Jin[2]†, Zhengong Meng[3]†, Hang Shan Choy[1], Zhixiang Cai[1], Guijun Li[1]*, Cheuk Lam Ho[3]*, Jinlong Yang[2], Wai Yeung Wong[3]*

[1]Department of Industrial and Systems Engineering, The Hong Kong Polytechnic University, Hung Hom, Hong Kong, HKSAR Email: mitch.li@polyu.edu.hk

[2]Hefei National Laboratory for Physical Sciences at Microscale, University of Science and Technology of China, Hefei, Anhui 230026, China

† These authors contributed equally.

[3]Institute of Molecular Functional Materials and Department of Chemistry, The Hong Kong Baptist University, Waterloo Road, Hong Kong, HKSAR, Email: clamho@hkbu.edu.hk, rwywong@hkbu.edu.hk

[4]School of Reliability and Systems Engineering, Beihang University, No. 37 Xueyuan RD. Haidian, Beijing 100191, China

This file includes:

Movie S1: Laser writing on malachite painting.
